# Editorial: Psychosocial, behavioral, and clinical implications for public mental health during the COVID-19 pandemic

**DOI:** 10.3389/fpsyt.2023.1274588

**Published:** 2023-08-18

**Authors:** Zonglin He, Babatunde Akinwunmi, Wai-kit Ming

**Affiliations:** ^1^Division of Life Science, Hong Kong University of Science and Technology, Kowloon, Hong Kong SAR, China; ^2^Brigham and Women's Hospital, Harvard Medical School, Boston, MA, United States; ^3^Department of Infectious Diseases and Public Health, Jockey Club College of Veterinary Medicine and Life Sciences, City University of Hong Kong, Kowloon, Hong Kong SAR, China

**Keywords:** COVID-19, mental health, psychosocial changes, public health, behavioral changes

The emergence of COVID-19 has caused widespread confusion, anxiety, and fear among the general public, which affected everyone worldwide. Exploring behavioral and psychosocial changes during the pandemic may shed light on the social determinants of mental disorders. It may prepare societies for novel mental health promotion and prevention interventions during future emerging disease outbreaks.

This Research Topic gathers diverse contributions highlighting the following topics: (1) the behavioral and psychosocial changes during the COVID-19 pandemic in various populations, (2) the preventive measures and governance enacted during COVID-19. This Research Topic consists of ten original articles, two systematic reviews, and three brief research reports.

The COVID-19 pandemic has profoundly impacted people's mental health worldwide. The research in this Research Topic sheds light on the various aspects of this impact, including the mental health of working women, healthcare professionals, university students, and the general public.

The infection may lead to psychiatric symptoms. Individuals infected with SARS-CoV-2 might experience persistent long-COVID symptoms more than 4 weeks post-recovery. Marchi et al. conducted a systematic review with 32 reports on patients with long COVID syndrome and found that the most common psychiatric symptoms are sleep disturbances, depression, post-traumatic symptoms, anxiety, and cognitive impairment.

Society has undergone significant transformations during COVID-19. Du et al. conducted a longitudinal study to assess the extent of stress, anxiety, and depression in Chinese residents at four different time points, from the initial outbreak to 26 months later. The study found that the respondents experienced high levels of stress, anxiety, and depression. Over time, stress symptoms decreased in the short term, while anxiety and depression symptoms remained unchanged.

Moreover, Aguayo et al. took advantage of the Predi-COVID study to explore the association between pre-existing psychological disorders during the COVID pandemic and the severity of COVID-19 infection, using the regular psychotropic medication use as an indicator for the severity of mood disorders. Their study concluded that pre-existing use of psychotropic medication is associated with more severe symptoms and prolonged recovery.

Han et al. developed a moderated mediation model based on the survey conducted on 536 employees in a Chinese company. They found that COVID-related work changes would negatively impact their mental health and boost their interpersonal conflict and aggression in working populations by increasing their ego depletion. Moreover, Akbar and Ghazal conducted quantitative, cross-sectional survey-based research involving over 300 female employees and students. They reported that in Pakistan, women faced increased occurrences of sexual harassment post-COVID-19, in addition to employment instability, both of which negatively affected their mental health.

Various public health strategies and mitigation measures have been implemented within society to control the pandemic. However, these measures have unavoidably led to mental health issues. Hui et al. conducted an online cross-sectional study on the family members of nursing home residents in China and reported that around one-quarter of family members had symptoms of anxiety owing to the restrictions to nursing home visiting, the extent of which is inversely associated with their satisfaction with the care quality.

Additionally, this Research Topic highlights the impact of COVID-19 on healthcare workers and their coping strategies. It emphasizes the need for appropriate support and resources to help healthcare workers deal with the emotional stress and burnout associated with pandemic control.

Zhao et al. conducted a cross-sectional study of Shanghai residents in China and utilized latent class analysis to identify the subgroups based on depressive symptoms, and they reported that medical staff, especially those with longer internet usage time and occurrence of daytime dysfunction, tended to have higher rates of depressive symptoms. Nurses are at the frontline when fighting COVID-19. Mao et al. recruited 740 female nurses using random cluster sampling to conduct an online cross-sectional study and found that 7.9 and 17.8% of the respondents exhibited symptoms of anxiety and depression, respectively. Moreover, they found that insomnia and post-traumatic experiences contribute to worsening symptoms while being married is a protective factor for depressive symptoms.

Furthermore, during the COVID-19 pandemic, Yang et al. conducted a cross-sectional study on 173 healthcare workers in the frontline district headquarters of COVID-19 pandemic control in Shenzhen, one of the largest metropolitans in China. They reported that 47.40% of frontline health workers manifest with burnout, a state of high emotional exhaustion or depersonalization, and their ways of coping with emotional negatives matter. Notably, a systematic review by García-Iglesias et al. reported that during the COVID-19 pandemic, suicidal thoughts were reported by 2.4–21.7% of healthcare workers, with 0.5–12.6% having attempted at least one-lifetime suicide and 0.5–3.5% having a recent suicide attempt.

The research papers in the Research Topics also call for appropriate mental healthcare preventive measures, policies, and governance during pandemics. Li et al. recruited around 3,000 university students in Shangdong Province, China, and conducted a longitudinal study to evaluate the impact of preventive behaviors on mental health. Their findings suggested that students are more tended to have depression and less likely to develop anxiety and stress in the ongoing COVID-19 pandemic, and importantly, those who actively practiced preventive behaviors such as mask-wearing, social distancing, and frequent hand washing were less likely to have mental health disorders. In this sense, compliance to practice preventive measures are important for not only the mitigation of the pandemic but also the maintenance of the mental health of the residents. Hong et al. examined the psychometric properties of a questionnaire regarding adherence to protective measures such as social distancing and health beliefs among Korean populations. Structural equation models were used to indicate the constructed relationships between health beliefs, viral anxiety, depression, and personal injunctive norms.

Moreover, this Research Topic also highlights the impact of COVID-19 on social factors. On the one hand, the COVID-19 may aggravate the pre-existing social discrepancies. For example, Turpin et al. utilized a longitudinal national study on mental health and assessed the associations between pandemic distress and racial issues. They used the segregation index and COVID racial bias scale to indicate the social segregation and extent of racial bias, and they found that the pandemic stress is positively associated with higher social segregation and racial bias. In contrast, higher social status and social support were associated with lower pandemic distress. Moreover, Chung et al. surveyed 1,018 middle-school students in Hong Kong SAR, China, using a maximum variation sampling of 12 secondary schools. They found that socioeconomic status is linked with worsening psychosocial wellbeing during the pandemic, and those with lower resilience have a more robust effect size. On the other hand, during the pandemic, the delay or absence of tackling of the social issues by governmental entities may also be to blame. Diaz-Castro et al. conducted an analytical qualitative study to analyze the governance processes in formulating healthcare policies specifically for those with mental disorders during the COVID-19 pandemic in Mexico using the governance analytical framework. This study found that the needs of people with mental health disorders were neglected in Mexico and measures should be taken to call for actions in the entire society.

Overall, the research in this topic addresses the mental health and psychosocial wellbeing in the context of pandemic, underscoring the importance of promoting appropriate policies, resources, and support to mitigate their negative impact on individuals and society as a whole.

## Author contributions

ZH: Conceptualization, Writing—original draft, Writing—review and editing. BA: Writing—original draft, Writing—review and editing. W-kM: Writing—original draft, Writing—review and editing.

